# New advances in beta-blocker therapy in heart failure

**DOI:** 10.3389/fphys.2013.00323

**Published:** 2013-11-14

**Authors:** Vincenzo Barrese, Maurizio Taglialatela

**Affiliations:** ^1^Section of Pharmacology, Department of Neuroscience, University of Naples Federico IINaples, Italy; ^2^Department of Medicine and Health Sciences, University of MoliseCapobasso, Italy

**Keywords:** beta blockers, heart failure, clinical trials as topic, elderly patients, pharmacogenomics

## Abstract

The use of β-blockers (BB) in heart failure (HF) has been considered a contradiction for many years. Considering HF simply as a state of inadequate systolic function, BB were contraindicated because of their negative effects on myocardial contractility. Nevertheless, evidence collected in the past years have suggested that additional mechanisms, such as compensatory neuro-humoral hyperactivation or inflammation, could participate in the pathogenesis of this complex disease. Indeed, chronic activation of the sympathetic nervous system, although initially compensating the reduced cardiac output from the failing heart, increases myocardial oxygen demand, ischemia and oxidative stress; moreover, high catecholamine levels induce peripheral vasoconstriction and increase both cardiac pre- and after-load, thus determining additional stress to the cardiac muscle (1). As a consequence of such a different view of the pathogenic mechanisms of HF, the efficacy of BB in the treatment of HF has been investigated in numerous clinical trials. Results from these trials highlighted BB as valid therapeutic tools in HF, providing rational basis for their inclusion in many HF treatment guidelines. However, controversy still exists about their use, in particular with regards to the selection of specific molecules, since BB differ in terms of adrenergic β-receptors selectivity, adjunctive effects on α-receptors, and effects on reactive oxygen species and inflammatory cytokines production. Further concerns about the heterogeneity in the response to BB, as well as the use in specific patients, are matter of debate among clinicians. In this review, we will recapitulate the pharmacological properties and the classification of BB, and the alteration of the adrenergic system occurring during HF that provide a rationale for their use; we will also focus on the possible molecular mechanisms, such as genetic polymorphisms, underlying the different efficacy of molecules belonging to this class.

## Introduction

The use of β-blockers (BB) in heart failure (HF) has been considered a contradiction for many years. Considering HF simply as a state of inadequate systolic function, BB were contraindicated because of their negative effects on myocardial contractility. Nevertheless, evidence collected in the past years have suggested that additional mechanisms, such as compensatory neuro-humoral hyperactivation or inflammation, could participate in the pathogenesis of this complex disease. Indeed, chronic activation of the sympathetic nervous system, although initially compensating the reduced cardiac output from the failing heart, increases myocardial oxygen demand, ischemia and oxidative stress; moreover, high catecholamine levels induce peripheral vasoconstriction and increase both cardiac pre- and after-load, thus determining additional stress to the cardiac muscle (Kubon et al., 2011). As a consequence of such a different view of the pathogenic mechanisms of HF, the efficacy of BB in the treatment of HF has been investigated in numerous clinical trials. Results from these trials highlighted BB as valid therapeutic tools in HF, providing rational basis for their inclusion in many HF treatment guidelines. However, controversy still exists about their use, in particular with regards to the selection of specific molecules, since BB differ in terms of adrenergic β-receptors (β-ARs) selectivity, adjunctive effects on α-receptors, and effects on reactive oxygen species and inflammatory cytokines production. Further concerns about the heterogeneity in the response to BB, as well as the use in specific patients, are matter of debate among clinicians. In this review, we will recapitulate the pharmacological properties and the classification of BB, and the alteration of the adrenergic system occurring during HF that provide a rationale for their use; we will also focus on the possible molecular mechanisms, such as genetic polymorphisms, underlying the different efficacy of molecules belonging to this class.

## History, structure and classification of adrenergic beta receptors

To date, three subtypes of β-receptors (β 1-AR, β 2-AR and β 3-AR) have been identified; the existence of a fourth subtype, called β 4, has been proposed to explain the sympathomimetic effects of some atypical β-agonists (such as CGP-12177) known to be inactive on the “classical” β-receptors; however recent evidence suggest that the putative β 4 receptor is more likely a novel functional state of β 1 receptor (Granneman, 2001).

β-ARs display peculiar tissue distribution and pharmacological properties: β 1 is the “cardiac” receptor, while β 2 is expressed predominantly in smooth muscle cells, and β 3 in the adipose tissue. Structurally, β-ARs belong to the family of G protein-coupled receptors, with seven transmembrane domains, an extracellular N-terminal region, and an intracellular C-terminus. Although, in the heart, β 1 is the predominant AR subtype, cardiac cells express also β 2 and, to a less extent, β 3, with quantitative differences depending on age and pathological conditions. In particular, β-AR population of the non-failing heart is composed of β 1 and β 2, in a ratio of 8:2; however, in both ageing and HF, the proportion of β 1 subtypes decreases due to mRNA down-regulation, while levels of β 2-AR remain stable, thus achieving a 1:1 ratio of β 1- and β 2-ARs (Lohse et al., 2003). In the heart, stimulation of β-ARs pathway leads to the activation of the Gs protein, with consequent stimulation of the adenylate cyclase, increase in intracellular cAMP, and protein kinase A-dependent phosphorylation and modulation of the activity of important proteins involved in myocardial contractility, such as L-type Ca^2+^ channels, troponin I and sarcoplasmic reticular Ca^2+^/ATPase inhibitory protein (Wachter and Gilbert, 2012). Moreover, through the β γ-subunits of the heterotrimeric G-protein, β-AR stimulation activates members of the G-protein receptors kinase family, named βARK (βγ-AR kinase) or GRK2 (G-protein receptor kinase 2), which are a primary mechanism of self-regulation of adrenergic stimulation. Indeed, by phosphorylating residues in the C-terminal region of the receptor, GRK2 induce the binding of protein such as β-arrestin to the receptor, thus causing its uncoupling from the Gs protein and its transductional pathways; moreover, GRK2-induced phosphorylation also cause an increase in the affinity of β-AR for the inhibitory G protein Gi, thus accelerating receptor desensitization (Rengo et al., 2012b).

Although the main intracellular pathway activated by β-ARs is Gs signaling, it has been demonstrated that stimulation of β-ARs can also activate Gi proteins, MAP kinases and other proteins involved in the control of cell cycle and apoptosis (Bogoyevitch et al., 1996), in a subtype-specific manner, thus differentially influencing cardiomyocytes fate. These evidence suggest that modulation of β-ARs can impact cardiac pathophysiology in different and multiple ways, well beyond than simply controlling heart mechanics.

## Molecular mechanisms underlying β adrenergic system dysregulation in HF and the efficacy of β-blockers

HF is associated to dramatic changes in neurohormonal balance. To compensate the reduced cardiac output, an increased activity of the adrenergic nervous system (ANS), as well as an activation of the Renin-Angiotensin-Aldosterone System (RAAS) occur. As a consequence of ANS hyperactivity, norepinephrine and epinephrine plasma levels increase, due to adrenal gland secretion and adrenergic terminals spillover, thus leading to chronic sympathetic stimulation of the heart. Such stimulation of cardiac β-ARs increases oxygen demand and myocardial work, thus contributing to cardiac muscle stress. In adult rat myocytes, β 1-AR mediates apoptotic signaling, whereas the β 2 subtype seems to stimulate antiapoptotic pathways coupling to the inhibitory G protein (G_i_) (Bristow, 2000). Stimulation of renal iuxtaglomerular β 1-AR can also activate RAAS, thus causing an increase in angiotensin-related cardiac remodeling and apoptosis (Lymperopoulos et al., 2013). Negative effects of ARs hyperactivity are also recapitulated by transgenic mouse models; indeed, transgenic mice overexpressing β 1-AR (Engelhardt et al., 1999) or cardiac Gαs (Iwase et al., 1996) show a cardiomyopathy with ventricular dilatation and systolic dysfunction; by contrast, cardiac overexpression of β 2-AR improves contractility in the healthy heart (Akhter et al., 1997). Chronic ANS activation is also responsible for the selective down-regulation of β 1-AR and for the functional uncoupling of both β 1- and β 2-ARs from their intracellular coupling mechanisms; these events are determined by activation of GRKs, particularly GRK2. Indeed, it has been demonstrated that, in chronic HF, GRK2 is up-regulated in cardiomyocytes, thus leading to a reduced responsiveness of the cardiac muscle to catecholamines stimulation (Rengo et al., 2011). GRK2-induced ARs uncoupling and down-regulation also occur in the adrenal gland, where the inhibitory feedback on catecholamine release mediated by inhibitory α2-ARs is reduced, thus determining an increase of circulating epinephrine (Lymperopoulos et al., 2007). GRK2 inhibition by a small peptide (β ARK ct) increases cardiac contractility, normalizes neurohormonal axis activity, and improves survival in several animal models of HF (Rockman et al., 1998).

The molecular evidence here briefly reviewed have reinforced the hypothesis that the changes in the ANS during HF are not merely adaptive, but rather play a direct pathogenetic role, opening novel avenues to interpret the efficacy of drugs acting by blocking β-ARs in HF. In fact, BB might exert beneficial effects well beyond those exerted in cardiac muscle (limiting AR decrease in number and functional desensitization) (Iaccarino et al., 1998), such as counteracting some aberrant maladaptive responses of ANS and RAAS neurohormonal systems occurring in HF. Indeed, it has been recently demonstrated that bisoprolol normalized the adrenal catecholamine production by reducing GRK2 levels, thus restoring the negative feedback on epinephrine release exerted by α2-AR (Rengo et al., 2012a). In addition, atenolol and bisoprolol treatment have been also shown to improve myocardial perfusion by enhancing neoangiogenesis in the failing heart, via activation of VEGF signaling pathway (Dedkov et al., 2005; Rengo et al., 2013).

## Beta-blockers: classification and pharmacology

BB are a wide and heterogeneous group of molecules acting as competitive and reversible antagonists of β-ARs. In addition to their ability to block β-ARs-signaling, BB show a variety of adjunctive actions that are often used as criteria for their classification (see Table 1).

**Table 1:** **Main pharmacological properties of BB**.

**Drug**	**Selectivity**	**ISA**	**α –AR blockade**	**Membrane-stabilizing activity**	**Bioavailability (%)**	**Half-life (hrs)**
**β non-selective**						
Propranolol	0	0	0	++	25	3–5
Nadolol	0	0	0	0	35	10–20
Timolol	0	0	0	0	50	3–5
Pindolol	0	++	0	±	75	3–4
Labetalol	0	+	+	±	20	4–6
Carvedilol	0	0	+	0	30	7–10
**β 1-selective**						
Metoprolol	++	0	0	±	40	3–4
Atenolol	++	0	0	0	50	5–8
Esmolol	++	0	0	0	−	0.13
Acebutolol	+	+	0	+	40	8–12 (diacetolol)
Bisoprolol	++	0	0	0	90	9–12
Nebivolol	++	0	0	0	12–96^*^	10–30^*^

Abbreviations: ISA: intrinsic sympathomimetic activity; α-AR: alpha adrenergic receptor.

* Depending on CYP polymorphisms.

Data are mainly from refs. (Rockman et al., 1998) and (Goodman and Gilman's, 2011).

BB can be classified according to:

### β1-AR-selectivity

Propranolol and timolol are the prototypical non-selective, “first generation” BB, showing the same affinity for both β 1 and β 2 subtypes. Subsequent research, aiming to find cardioselective drugs, led to the synthesis of molecules such as atenolol, bisoprolol and metoprolol, preferentially blocking “cardiac” β 1-AR.

### α-AR-antagonism and vasodilator activity

Labetalol, bucindolol and carvedilol also act as α1-AR-antagonists. This pharmacological effect is particularly important in HF, since the peripheral vasodilatation induced by α1-AR blockade decreases both pre- and after-loads, thus reducing myocardial oxygen consumption and work. Other BB such as nebivolol, although not provided of α1-AR antagonism, show marked vasodilator properties; possible mechanisms explaining this effect are: stimulation of nitric oxide production, blockade of Ca^2+^ entry, opening of K^+^ channels, and antioxidant activity. In particular, nebivolol has been demonstrated to activate endothelial β 3-AR, thus stimulating NO production and dilation of coronary arteries (Rozec et al., 2006).

### Intrinsic sympathomimetic activity (ISA)

Although classified as blockers, some molecules belonging to this class can act as partial agonists, thus activating β 1-AR. The intrinsic sympathomimetic activity of pindolol, acebutolol and celiprolol can improve BB tolerability, since they do not cause severe bradycardia or excessive negative inotropic effects at rest. On the other hand, some BB also act as inverse agonists, thus inducing a negative response of receptor signaling in absence of the natural agonist. This characteristic has clinical consequences, since drugs as bucindol (with low inverse agonist activity) decrease mean and peak heart rate, while they do not reduce minimum heart rate (Wachter and Gilbert, 2012).

### Pharmacokinetics

BB widely differ in their physico-chemical properties. In particular, lipophilic compounds such as metoprolol, bucindolol, carvedilol and nebivolol, when given orally, are rapidly adsorbed in the gastrintestical tract and are extensively metabolized by the liver, therefore often presenting with a shorter half-life when compared to other BB. Moreover, the high lipophilicity and the resulting higher penetration across the blood-brain barrier could also explain the increased number of brain-related adverse events, as well as the membrane-stabilizing (quinidine-like) properties of antiarrhythmic molecules which appear independent of their BB activities (Murray et al., 1990).

### Other properties

Carvedilol and its metabolites are also endowed with antioxidant activity, a property that can be useful in the treatment of HF; propanolol and carvedilol seem to decrease vascular smooth muscle cells proliferation. Moreover, carvedilol and bucindolol can modulate guanine-nucleotide binding to its receptor (Bristow et al., 1992). It has been also demonstrated that carvedilol ameliorates insulin sensitivity (Jacob et al., 1996).

## Pharmacokinetics

As stated before, lipophilicity is one of the chemical characteristics influencing bioavailability and, consequently, administration schedule. High lipophilic molecules such as propanolol are rapidly adsorbed but, at the same time, they become extensively metabolized by the liver (first-pass metabolism); by contrast, drugs with intermediate lipophilic properties (bisoprolol) are efficiently adsorbed in the gut but poorly removed by the liver first-pass metabolism, so that they display high bioavailability (90%). Many BB are metabolized by the liver through the CYP pathway, in particular via the CYP2D6 isoform; in humans, the CYP2D6 enzyme is highly polymorphic and this characteristic has been often referred to explain the inter-individual variability observed in the plasma levels of drugs such as carvedilol and in the responses to treatment with BB in HF. Other CYP isoforms (CYP1A2, CYP2C9, CYP2C19 and CYP3A4) can contribute, though to a lesser extent, to BB hepatic metabolism, while other molecules, such as bisoprolol, are excreted unmodified via the kidney. As a consequence, dose modification of BB should be considered in patients with pathological conditions impairing both liver and kidney functions, or for patients treated also with drugs metabolized by the same CYP isoforms (antidepressants, antipsychotics). Moreover, it should be mentioned that peak plasma concentrations, as well as half-lives, are strongly influenced by the formulation of the molecule; this has important consequences on clinical outcomes. A paradigmatic example is represented by the two different formulations of metoprolol used in controlled clinical trials; indeed, in the COMET study (Carvedilol or Metoprolol European Trial), the reduced efficacy demonstrated in the metoprolol-tartrate arm with respect to the carvedilol arm has been largely explained by the shorter half-life of metoprolol-tartrate when compared to carvedilol. It has been suggested that this pharmacokinetic difference led to a different degree of beta blockade in patients enrolled in the two study arms, and, therefore, that the dose of metoprolol tartate administered in this study was inadequate (Poole-Wilson et al., 2003). In a subsequent trial, MERIT-HF (Metoprolol CR/XL Randomized Intervention Trial in Congestive Heart failure), a different and longer-lasting formulation, metoprolol-succinate, was demonstrated to reduce mortality and hospitalization in HF patients (Hjalmarson et al., 2000); the degree of these protective effects were now similar to those demonstrated for other BB with longer plasma half-lives. Based on these evidence, FDA has approved metoprolol-succinate, but not metoprolol-tartrate, for the treatment of patients with HF.

## Tolerability of β-blockers

The most common adverse events of BB result from their mechanism of action. The blockade of sympathetic stimulation may have both acute or chronic consequences, mainly giving rise to symptoms of cardiovascular, respiratory, central nervous system and metabolic origins.

In particular, acute blockade of catecholamines effects can induce bradycardia that can be potentially life-threatening in patients with defects in atrio-ventricular conduction; moreover, acute blockade of β-ARs can worsen myocardial contractility and, consequently, induce or deteriorate HF in patients with myocardial infarction, cardiomegaly or compensated HF. Starting with low doses and slowly titrating up (in order to reach the optimal dose in several weeks) is a widely used and well known strategy to reduce these risks. In the same vein, given that a prolonged BB treatment may induce β-ARs up-regulation and, consequently, an enhanced sensitivity to catecholamines, abrupt withdrawal of BB should be avoided in order to prevent angina and risk of sudden death. BB treatment can also induce Raynaud's phenomenon and worsen peripheral vascular disease.

As β2-ARs-blockade cause bronchoconstriction, BB have been considered as contraindicated in patients suffering from asthma or Chronic Obstructive Pulmonary Disease (COPD). Thus, concerns exist regarding the use of BB in patients with HF and COPD, in particular for non-cardioselective molecules; however, the risk to increase bronchoconstriction, thus worsening COPD symptoms, is often overcome by the beneficial effects on HF. For these reasons, COPD is currently considered as a relative contraindication for BB treatment, and a careful risks-benefits assessment should be made to avoid undertreatment of HF patients with respiratory comorbidities (Ellison and Gandhi, 2005).

BB also impact glucose homeostasis, since catecholamines, mainly through β 2-AR, promote glycogenolysis and glucose-mobilization in response to hypoglycemia, thus increasing blood glucose levels. Therefore, BB can conceal symptoms of hypoglycemia or cause an excessive reduction of blood glucose in susceptible diabetic patients treated with insulin or oral hypoglycemic agents. β 1-AR selective antagonists, as well as carvedilol, the latter shown to improve insulin-sensitivity, could possibly represent a valid alternative for HF patients with concomitant diabetes.

## Efficacy of BB in HF

The efficacy of BB in the treatment of HF has been evaluated in several randomized, controlled clinical trials (Table 2). Patients enrolled in these studies suffered from HF with different etiology and showed an impaired systolic function. Taken together, the aforementioned clinical trials have demonstrated that three BB (metoprolol succinate, bisoprolol and carvedilol) are able to improve ventricular ejection fraction and HF symptoms, and to reduce mortality and hospitalizations. Of note, numerous trials were prematurely discontinued for mostly ethical reasons, since interim analysis showed a reduced mortality in the BB arm compared to placebo-arm. Bucindolol and nebivolol have also been tested in clinical trials, with different results.

**Table 2:** **Summary of main clinical trials reported in the text investigating the efficacy of BB in HF**.

**Study name (reference)**	**No patients**	**BB used**	**Description**	**Main findings**	**Additional findings and comments**
MDC (Waagstein et al., 1993)	383	Metoprolol	HF secondary to Dilated Cardiomyopathy (EF <40%)	34% decrease in mortality or need for transplantation	No significant difference in mortality alone
MERIT-HF (Hjalmarson et al., 2000)	3991	Metoprolol succinate	Mild-moderate HF (EF <40%) NYHA II-IV	<34% decrease in all cause mortality	<39% decrease in cardiac death and non-fatal MI
CIBIS (CIBIS Investigators and Committees, 1994)	641	Bisoprolol	Moderate HF (EF <40%) NYHA III-IV	No significant difference in mortality	Significant improvement of functional status of the patients
CIBIS II (CIBIS-II Investigators, 1999)	2647	Bisoprolol	Moderate HF (EF <35%) NYHA III-IV	32% decrease risk of mortality and hospitalization for HF	Greatest effects in patients with ischaemic HF and NYHA III at baseline
CIBIS III (Willenheimer et al., 2005)	1010	Bisoprolol (vs. enalapril)	Mild moderate HF (EF <35%) NYHA II-III	Non-inferiority of bisoprolol vs enalapril in reducing mortality as first treatment in ITT	Non-inferiority of bisoprolol was not proven in *per-protocol* analysis
US Carvedilol study (Packer et al., 1996)	1094	Carvedilol	Mild moderate HF NYHA II-IV	65% mortality reduction	38% reduction in death or hospitalization for cardiovascular reasons
COPERNICUS (Packer et al., 2002)	2289	Carvedilol	Severe HF (EF <25%) NYHA III-IV	35% in risk of death	27% decrease death or hospitalization for a cardiovascular reason
CAPRICORN (The CAPRICORN Investigators, 2001)	1959	Carvedilol	Patients with recent MI and left ventricular dysfunction (EF <40%)	23% reduction in mortality	No significant difference in primary endpoint (all-cause mortality or hospitalization for cardiovascular problems)
COMET (Poole-Wilson et al., 2003)	3029	Carvedilol vs. Metoprolol tartrate	Mild moderate HF (EF <35%) NYHA II-IV	17% decrease in carvedilol- vs. metoprolol- arm	Concerns about metoprolol formulation
BEST (BEST Investigators, 2001)	2708	Bucindolol	Mild moderate HF (EF <35%) NYHA III-IV	No significant overall survival benefit	Reduction in mortality in patients homozygous for Arg389 (subsequent pharmacogenetic analysis)
SENIORS (Flather et al., 2005)	2128	Nebivolol	Mild moderate HF (EF <35% in last 6-months) Age >70yrs	14% reduction mortality and hospitalizations	Significant increase of LVEF and decrease in end-systolic volume

The Table re-elaborates and integrates the data reported in Table I of Kubon et al., 2011.

### Metoprolol

Metoprolol has been one of the first BB to be studied in HF treatment. 383 patients with systolic disfunction secondary to dilated cardiomyopathy were randomized to placebo or metoprolol at a target dose of 100–150 mg/daily. Metoprolol was shown to reduce mortality and need of transplantations by 34% with respect to placebo arm (Waagstein et al., 1993). A subsequent wider trial (3991 patients in 14 countries), MERIT-HF, investigated the efficacy of metoprolol succinate in mild-moderate HF patients with impaired systolic function (left ventricular ejection fraction-LVEF <40%) and New York Heart Association (NYHA) functional class II–IV; in this study, a long-lasting release metoprolol formulation was used, with a mean daily dose of 159 mg (Hjalmarson et al., 2000). The authors found a 34% statistically-significant decrease in mortality and a reduction in combined endpoint “all-cause mortality and hospitalization” (risk reduction by 19%); the beneficial effects of metoprolol were even higher on cardiac death and non-fatal acute myocardial infarction, with a 39% risk reduction.

### Bisoprolol

Following a previous Cardiac Insufficiency Bisoprolol Study (CIBIS), in which bisoprolol administration showed a non-statistically significant trend toward improved survival in HF patients (1994), a subsequent CIBIS-II trial enrolled a higher number of subjects (*n* = 2647) with LVEF <35% and NYHA class III–IV. As for metoprolol, a reduction in all-cause mortality was demonstrated for bisoprolol-treated patients when compared to placebo-arm (11.8 vs. 17.3%, respectively), with a follow-up of about 1 year (1999). More recently, a third study with bisoprolol (CIBIS III) has addressed the relevant issue of whether BB could be as effective as renin-angiotensin-aldosterone system (RAAS) inhibitors as first-line drugs in HF. In general, therapy in CHF patients is initiated with an ACE inhibitor or an AngII-receptor blocker (ARB), and thereafter a β-blocker is introduced. This drug sequence seems to result largely from the fact that, historically, the beneficial effects of ACE inhibitors were documented first. The order of initiation of these agents is of great relevance, because the first agent initiated is more likely to be titrated up to its target dose, whereas the second agent is often given at a suboptimal dose or not initiated at all. Theoretically, sympathetic system alterations occur before RAAS dysfunction during chronic HF; moreover, it is well known that stimulation of β 1-ARs in iuxtaglomerular cells can increase renin production, thus contributing to RAAS over activation. The CIBIS III trial compared bisoprolol to enalapril, one of the most used first-line drug for HF. Patients with LVEF <35% were randomized to bisoprolol- or enalapril; after a 6-months mono-therapy period, patients received both drugs. The results obtained showed the non-inferiority of bisoprolol-first versus enalapril-first strategy, in the Intention to treat population but not in per-protocol analysis, thus requiring more data to better clarify this issue (Willenheimer et al., 2005).

### Carvedilol

This non-selective, third generation BB has been tested in different trials. The first randomized study evaluated the efficacy of carvedilol on 1094 US patients with LVEF <35%, stratified according to their performance on exercise tests. Carvedilol was shown to reduce mortality risk by 65% when compared to placebo; these data induced the Monitoring Board to terminate the study before its scheduled completion (Packer et al., 1996). The efficacy of carvedilol has been evaluated also in patients with severe HF, a group often not included in randomized trials. The COPERNICUS study enrolled 2289 patients with symptoms of HF at rest or on minimal exertion and with LVEF <25%, randomized to carvedilol or placebo; in the carvedilol arm, a reduction by 35% in the risk of death and an improvement in HF symptoms were observed (Packer et al., 2002). Carvedilol has been evaluated also in patients with left ventricular dysfunction, with or without HF, secondary to myocardial infarction. The aim of the CAPRICORN study was to evaluate whether addition of carvedilol to standard modern management of acute myocardial infarction would improve outcome in terms of mortality and morbidity. The results obtained confirmed that carvedilol decreased the risk of mortality by 23% when compared to placebo (2001).

It should be mentioned that clinical trials have investigated efficacy of BB in patients with impaired LVEF (<40%); however, since BB lower myocardial work by reducing heart rate and oxygen demand, it seems desirable to extend these studies also to HF patients with preserved systolic ejection fraction such as the elderly population.

## Comparison between BB

Collectively, results emerged from clinical trials demonstrated that blockade of β-AR signaling has beneficial effects in HF, both reducing mortality and improving symptoms of impaired cardiac function. Such protection has been demonstrated for different molecules belonging to BB class, and the degree of the beneficial effects was quite similar for any of the chosen drug. Thus, the possibility existed that BB efficacy in HF was due to a “class effect.” On the other hand, many researchers have pointed out that peculiar pharmacodynamic and/or pharmacokinetic properties (β 1-AR-selectivity, vasodilating actions, anti-oxidant activity, “pleiotropic effects”, good bioavailability, low drug-drug interaction risk) could represent valuable characteristics for preferring a specific molecule over other BBs in the treatment of HF. To verify this hypothesis, a specific clinical trial was conducted in 2003. The COMET study aimed to compare the mortality risk-reducing effects in 3029 patients with HF randomized to receive either carvedilol or metoprolol, after a mean follow-up of 58 months. The results demonstrated that carvedilol decreased mortality by 17% with respect to metoprolol (Poole-Wilson et al., 2003); however, as mentioned before, subsequent analyses showed that the degree of β-blockade reached with the formulation of metoprolol used in the study (metoprolol tartrate) was not adequate, and probably not similar to that of the carvedilol arm. Basically, metoprolol tartrate should have been titrated up in the COMET study (Talber, 2004).

A solution to this issue has been also pursued by several meta-analyses; most of these have been focused on carvedilol, possibly because of its unique pharmacodynamic profile. Nevertheless, results from these studies seem to be conflicting; in a systematic review of 11 randomized controlled trials in 5,207 patients, it was found that carvedilol reduced all-cause mortality in patients with HF significantly more when compared to other BB such as atenolol, bisoprolol and metoprolol. According to the Authors, the superiority of carvedilol over other BB could be explained by the peculiar actions exerted by carvedilol, such as: antiarrhythmic effect (and consequent reduction in the risk of sudden death), a more sustained increase in LVEF when compared to other BB, blockade of up-regulated β 2-AR, vasodilation. (DiNicolantonio et al., 2013). By contrast, in their meta-analysis of 21 trials including 23122 HF patients, Chatterjee et al. found no significant differences in mortality outcome among the BB considered (atenolol, bisoprolol, bucindolol, carvedilol, metoprolol, and nebivolol), thus concluding that the beneficial of BB were due to a class effect. Nonetheless, carvedilol showed the lowest cardiac mortality among all β blockers tested, although this was not statistically significant; in consideration also of the its beneficial effects on lipid and glucose profile, the Authors suggest the use of carvedilol as an empiric initial treatment in patients with cardiovascular comorbidities (Chatterjee et al., 2013).

Lack of clear data from clinical trials regarding the superiority of a given BB are reflected in current guidelines for the management of HF, in which the use of a particular BB over the others is not specified.

## Pharmacogenomics of BB

As for many other pharmacological treatments, variability in clinical and functional outcomes exists also for BB administration in HF patients. Actually, BB treatment fails to improve LVEF in a variable proportion of subjects, while a minority of patients experience worsening of HF symptoms during BB titration (Talameh et al., 2012); such differences could be explained, at least in part, by genetic variation influencing BB pharmacodynamics and/or pharmacokinetics. To date, polymorphisms in β 1-AR, β 2-AR, GRK-5, CYP2C6, NET and UGT1A1 have been associated to variability in BB response.

### β1-AR

The most studied polymorphism in the β 1-AR gene (ADRB1) is the Arg389Gly. β 1-ARs possessing arginine residue show greater activity, both in basal condition and after agonist-stimulation. Therefore, in these patients, β-blockade might potentially have a greater effect by reducing the high sympathetic stimulation determined by the β 1-Arg receptor (Talameh and Lanfear, 2012). On the other hand, the high sympathetic activity could require higher dose of BB. The most convincing result from the literature concerning the possible role of Arg389Gly variant in BB treatment comes from BEST study, a trial investigating the effects of bucindolol in HF. Despite the main study failed to demonstrate the efficacy of bucindolol in all the population studied, subsequent pharmacogenetic analysis clearly demonstrated that bucindolol significantly reduced mortality when compared to placebo in patients homozygous for Arg389 (2001). Nevertheless, different studies, such as the pharmacogenetic sub-study of MERIT-HF, failed to find association between Arg389 allele and mortality outcome, suggesting that results from BEST depended on bucindolol peculiar properties (in particular to its marked ability to suppress β 1-ARs), and could not be extended to other BB.

Another pharmacogenetic sub-study, the HF-ACTION DNA, demonstrated that patients with the Arg/Arg genotype required a higher dose of BB to achieve a mortality risk reduction similar to that of Gly carriers (Fiuzat et al., 2013).

Another variant of ADRB1, Ser49Gly, has been associated to differential outcome in HF and response to β-AR antagonists. The presence of the glycine residue is thought to enhance agonist-promoted down-regulation of β 1-AR with respect to Ser49 (Levin et al., 2002), thus preserving failing myocardium from toxic effects exerted by catecholamines. However, data about its possible role in determining BB response are poor and inconsistent.

### β2-AR

As stated before, β 2-AR signaling plays a more relevant role in the failing heart, since β 1-AR expression is down-regulated. Thus, variants in ADRB2 gene could modulate response to BB treatment. Among the three polymorphisms identified in β 2-AR gene, Gln27Glu variation has been investigated; Glu27 β 2-AR shows an enhanced resistance to desensitization. Results from clinical trials are conflicting: some studies found a lower proportion of responders to BB treatment for patients homozygous for Gln27, other trials failed to find associations. However, these negative data could be explained by the reduced number of participants and by the inclusion of β 1-AR-selective blocker (Talameh et al., 2012).

### α2_C_-AR and GRK-5

Genetic variants of the presynaptic α2 receptor and of the G-protein coupled receptor kinase-5 seem to contribute to the variability in BB response. In particular, association of polymorphisms of α2_C_-AR and GRK-5 with the Arg389 variant in β 1-AR might impact response to BB. Indeed, a variant of the α2_C_-AR gene leading to a deletion of amino acids 322–325, which resulted in an increase of catecholamine stimulation, when associated to the Arg389 variant in the ADRB1, caused a more pronounced increase in LVEF with respect to other genotypes. In the same way, patients with the Gln41variant in GRK5 and Arg389 in β 1-AR showed advantages in term of reduced mortality after BB treatment (Talameh et al., 2012).

### CYP2D6 and UGT1A

As mentioned before, CYP2D6 is the main CYP isoform involved in BB metabolism. Thus, genetic variants in this gene have been suggested to modulate BB response. In the same vein, polymorphisms in UGT1A1 have been also proposed to modify BB metabolism and, consequently, response to pharmacological treatment. The study of (Baudhuin et al., 2010) retrospectively analyzed 93 patients characterized as responders or non-responders to metoprolol or carvedilol therapy. These patients have been also classified according to their genotype in different classes, ranging from poor to extensive metabolizer, for both CYP2D6 and UGT1A1 variants. The Authors did not find any association between CYP2D6 and UGT1A1 polymorphisms and response to therapy with carvedilol or metoprolol; nevertheless, patients who were poor metabolizer for CYP2D6 required a higher dose of carvedilol. These data might be relevant in patients also carrying ADRB1 Gly/Gly variant, since higher dose of carvedilol should be used to reach beneficial effects on HF (Baudhuin et al., 2010).

Taken together, these results, although suggestive of the possibility to select BB according to a particular genotype, are not conclusive, thus requiring large prospective clinical trials.

## BB in specific group of patients

Although their efficacy in HF has been demonstrated, BB are not given or are inadequately administered to some categories of patients. Among them, elderly patients are frequently undertreated, because of comorbidities, reduced tolerability to BB, and risk to worsen symptoms of HF. Moreover, elderly patients frequently show preserved LVEF, thus making even harder the decision to start BB therapy. Nevertheless, analysis of randomized trials have shown that BB reduce mortality and improve quality of life also in patients >70 years, as well as in younger ones. Among BB, nebivolol has been considered as the most interesting for HF in elderly because of its unique pharmacodynamic profile. Indeed, the β 1-AR-selective antagonist nebivolol is not provided with vasoconstrictor activity and should not interfere with respiratory function; moreover, stimulation of NO release might improve diastolic function. These characteristics might be relevant in elderly patients, frequently showing comorbidities and commonly less tolerant to peripheral vasoconstriction (Del Sindaco et al., 2010). Based on these evidence, two clinical trials have investigated efficacy of nebivolol in aged HF patients. While the ENECA study demonstrated an improvement in LVEF in patients >65 years (Edes et al., 2005), the SENIORS trial showed that nebivolol reduce mortality and hospitalizations by 14% when compared to placebo. Nebivolol was also well tolerated, including in patients with impaired renal function, and the proportion of patients discontinuing treatment due to adverse events was similar in nebivolol and placebo arms (Flather et al., 2005).

As stated before, COPD is considered a contraindication to BB treatment because of the risk to induce bronchoconstriction, thus worsening symptoms. To this aim, cardioselective BB have been proposed to overcome the possible lack of tolerability by HF patients with concomitant COPD. A recent analysis by Mentz et al. has compared β 1-selective versus non-selective drugs in determining worse outcomes in HF patients enrolled in the OPTIMIZE-HF trial. The Authors found no evidence that cardioselectivity was associated with better outcomes, in terms of both mortality and tolerability. The Authors also suggest that use of non-selective BB might be helpful for HF (antagonism on β 2-AR, whose signaling has a relevant role in the failing heart) and COPD (reduction of pulmonary desensitization caused by β 2-agonists). These data support the hypothesis that BB might be safe and well tolerated also in patients with HF and COPD (Mentz et al., 2013).

## Conclusions

BB use is strongly recommended in all current guidelines for patient with symptomatic HF and impaired systolic function, unless there is a contraindication. Although wide, randomized, controlled clinical trials have investigated efficacy of three molecules, thus driving to the registration of carvedilol, metoprolol succinate and bisoprolol for the treatment of HF, it appears reasonable to suppose that BB efficacy lies in their ability to counteract adrenergic overactivation, more than in additional, molecule-related properties. Such consideration is supported by different meta-analyses suggesting that BB efficacy should be considered as a class effect. However, peculiar mechanisms of action of distinct BB (i.e., vasodilation, NO release, anti-oxidant activity, anti-proliferant actions on vascular smooth muscle cells) might represent additional and useful tools for HF therapy and to increase treatment tolerability, mainly in selected groups of patients such as elderly; a careful evaluation of the patient and his clinical condition should be made. For instance, molecules such as carvedilol, which have been demonstrated to ameliorate insulin sensitivity, could be useful in patients with concomitant diabetes. Moreover, analysis of genetic polymorphisms in β-ARs and metabolic enzymes, might also contribute to find “the best drug to the best patient.”

In conclusion, BB are currently a cornerstone in HF therapy, and their use should be extended also to groups of patients commonly undertreated, such as elderly or comorbid patients.

### Conflict of interest statement

The authors declare that the research was conducted in the absence of any commercial or financial relationships that could be construed as a potential conflict of interest.
